# Desafios e Perspectivas dos Biomarcadores de Fibrose Cardíaca: Do Diagnóstico à Aplicação Clínica

**DOI:** 10.36660/abc.20240735

**Published:** 2025-01-22

**Authors:** Priscila Portugal dos Santos, Ricardo Luiz Damatto

**Affiliations:** 1 Universidade Estadual Paulista Júlio de Mesquita Filho Campus de Botucatu Faculdade de Medicina de Botucatu Botucatu SP Brasil Universidade Estadual Paulista Júlio de Mesquita Filho Campus de Botucatu – Faculdade de Medicina de Botucatu, Botucatu, SP – Brasil; 2 Faculdade de Ciências Sociais e Agrárias de Itapeva Itapeva SP Brasil Faculdade de Ciências Sociais e Agrárias de Itapeva, Itapeva, SP – Brasil

**Keywords:** Fibrose Endomiocárdica, Colágeno, Matriz Extracelular, Biomarcadores

Conforme relatado por Cheng-Mei et al.,^1^ a fibrose miocárdica está presente em diversas doenças cardíacas e pode ter impacto negativo no coração. Ela desempenha papel central na disfunção e falência cardíaca em várias patologias, além de ser forte indicador de desfechos clínicos desfavoráveis e de mortalidade.^2^ No entanto, é essencial reconhecer que a fibrose é um processo complexo e heterogêneo, que em algumas condições cardíacas pode ter papel importante, como após infarto do miocárdio. Nessa situação, a fibrose atua como um mecanismo reparador, substituindo cardiomiócitos mortos por tecido cicatricial. Essa resposta fibrótica inicial é fundamental para evitar a ruptura cardíaca, complicação grave e fatal, que é uma das principais causas de morte em pacientes com infarto agudo do miocárdio.^3^

A fibrose cardíaca caracteriza-se pelo acúmulo excessivo de componentes da matriz extracelular (MEC), especialmente colágenos, no miocárdio.^4^ É importante compreender que esse processo envolve mais do que apenas a quantidade de colágeno; a caracterização da fibrose inclui também a qualidade do colágeno, a proporção entre os diferentes tipos de fibras e o grau de *cross-linking* do colágeno.^5^ O colágeno tipo I, predominante na MEC cardíaca e vascular, contribui para a resistência e rigidez do tecido devido à sua estrutura espessa e densa. Já o colágeno tipo III, por ser mais flexível, promove a complacência da MEC. A elasticidade do miocárdio é, determinada pela proporção entre as fibras de colágeno tipo I e tipo III.^2,6^ Além disso, o grau de ligações covalentes entre as microfibrilas, ou seja, o *cross-linking*, é fator crítico na rigidez das fibras de colágeno, tornando-as mais resistentes à degradação e contribuindo para o enrijecimento do tecido.^2,7^ Portanto, para melhor compreensão do processo de fibrose cardíaca, e desenvolvimento de novas terapias e métodos diagnósticos, é fundamental avaliar a quantidade, a qualidade e o grau de *cross-linking* do colágeno.

Embora a fibrose miocárdica seja um elemento comum entre diversas doenças cardíacas, os métodos atuais para sua detecção e diagnóstico clínico ainda são limitados e carecem de melhorias quanto à sensibilidade e especificidade.^2^ Para avaliação clínica, a biópsia endomiocárdica (BEM) é considerada o padrão-ouro, permitindo uma análise direta e específica da deposição de colágeno por meio de técnicas histológicas, como coloração com picrosirius red ou tricrômico de Masson em amostras de biópsia. Apesar de o procedimento ter baixo risco de complicações graves, é invasivo e envolve desafios para a triagem de fibrose em grandes populações, além de limitações de representatividade, devido ao pequeno volume de tecido examinado e possíveis erros de amostragem.^2,8^

Parâmetros obtidos por técnicas de imagem apresentam certas vantagens em relação à BEM, sendo métodos não invasivos, capazes de avaliar o coração inteiro e adequados para monitoramento da progressão da doença e da resposta ao tratamento. Entretanto, os biomarcadores de imagem apresentam resolução relativamente baixa e uma capacidade limitada de discriminar características específicas do tecido.^2,5^

Nesse cenário, a análise de biomarcadores circulantes tem se destacado como estratégia promissora, que apresenta boa reprodutibilidade, pode ser implementado para grandes populações mais facilmente, e é independente do operador. Isso a torna útil para triagem populacional, acompanhamento da progressão da doença e eficácia terapêutica ao longo do tempo. Apesar de muitos biomarcadores terem sido propostos nos últimos anos, poucos apresentam resultados consistentes e validação robusta que sustentem sua implementação em ensaios clínicos e na prática clínica. É provável que a complexidade e heterogeneidade da fibrose miocárdica exijam o uso de painéis de múltiplos biomarcadores para melhorar a precisão diagnóstica.^2^ Nesse contexto, abordagens de bioinformática, como a utilizada no estudo,^1^ desempenham um papel essencial na triagem inicial e identificação de potenciais biomarcadores de fibrose cardíaca, enquanto técnicas de proteômica e peptidômica são fundamentais para validar esses candidatos e investigar sua aplicabilidade clínica.^2,9,10^

Outro aspecto relevante é analisar se a capacidade diagnóstica e preditiva desses biomarcadores, bem como a avaliação de custo-efetividade, em comparação com os biomarcadores convencionais,^11^ é melhor. Após essa etapa, é essencial propor novas tecnologias em saúde para que os avanços sejam efetivamente transferidos para a prática clínica em benefício dos pacientes.
