# Supplementary material for: Desafios e Perspectivas dos Biomarcadores de Fibrose Cardíaca: Do Diagnóstico à Aplicação Clínica
Source: Arq Bras Cardiol. 2025 Jan 22;121(12):e20240735. [Article in Portuguese] doi: 10.36660/abc.20240735 (PMC11809844; doi:10.36660/abc.20240735)
Supplement: Translation

# Translation: Challenges and Perspectives of Cardiac Fibrosis Biomarkers: From Diagnosis to Clinical Application

**DOI:** 10.36660/abc.20240735i

**Authors:** Priscila Portugal dos Santos, Ricardo Luiz Damatto

**Affiliations:** 1 Botucatu SP Brazil Universidade Estadual Paulista Júlio de Mesquita Filho Campus de Botucatu – Faculdade de Medicina de Botucatu, Botucatu, SP – Brazil; 2 Itapeva SP Brazil Faculdade de Ciências Sociais e Agrárias de Itapeva, Itapeva, SP – Brazil

**Keywords:** Endomyocardial Fibrosis, Collagen, Extracellular Matrix, Biomarkers

As reported by Cheng-Mei et al.^1^ myocardial fibrosis is present in various cardiac diseases and can negatively impact the heart. It plays a central role in cardiac dysfunction and failure across different pathologies, serving as a strong predictor of poor clinical outcomes and mortality.^2^ However, it is essential to recognize that fibrosis is a complex and heterogeneous process, which can sometimes play a beneficial role, such as after a myocardial infarction. In this context, fibrosis acts as a reparative mechanism, replacing dead cardiomyocytes with scar tissue. This initial fibrotic response is crucial to prevent cardiac rupture, a severe and often fatal complication and one of the leading causes of death in patients with acute myocardial infarction.^3^

Cardiac fibrosis is characterized by the excessive accumulation of extracellular matrix (ECM) components, especially collagens, within the myocardium.^4^ Understanding this process involves more than just collagen quantity; the characterization of fibrosis also considers collagen quality, the ratio of different fiber types, and the degree of collagen cross-linking.^5^ Type I collagen, which predominates in cardiac and vascular ECM, contributes to tissue strength and stiffness due to its thick, dense structure. Type III collagen, being more flexible, aids in ECM compliance. Thus, myocardial elasticity is determined by the ratio of type I to type III collagen fibers.^2,6^ Moreover, the degree of covalent cross-linking among the constituent microfibrils is critical to collagen fiber rigidity, increasing their resistance to degradation and significantly contributing to tissue stiffening.^2,7^ Therefore, a comprehensive understanding of cardiac fibrosis, aimed at developing new therapies and diagnostic methods, requires evaluating collagen quantity, quality, and cross-linking levels.

Although myocardial fibrosis is a common feature in various cardiac diseases, current methods for its clinical detection and diagnosis remain limited, with room for improvement in sensitivity and tissue specificity.^2^ For clinical evaluation, endomyocardial biopsy (EMB) is considered the gold standard, providing a direct, specific analysis of collagen deposition via histological techniques such as picrosirius red or Masson's trichrome staining on biopsy samples. Although EMB is associated with a low risk of serious complications, it is invasive and presents logistical challenges for fibrosis screening in large populations, with limitations in sample representativeness due to the small tissue volume analyzed and potential sampling erros.^2,8^

Parameters derived from imaging techniques offer certain advantages over EMB, being non-invasive, capable of assessing the entire heart, and suitable for monitoring disease progression and therapeutic response. However, imaging biomarkers have relatively low resolution and limited capacity for discriminating specific tissue characteristics.^2,5^

In this scenario, circulating biomarker analysis has emerged as a promising strategy, providing a reproducible, operator-independent, and scalable alternative for large populations. This approach is useful for population-wide screening, monitoring disease progression, and assessing therapeutic efficacy over time. Although numerous biomarkers have been proposed in recent years, few have accumulated consistent data and robust validation to support their implementation in clinical trials and routine clinical practice. Given the complexity and heterogeneity of myocardial fibrosis, it is likely that multiple biomarker panels will be needed to enhance diagnostic accuracy.^2^ In this context, bioinformatics approaches, such as those applied in the study,^1^ are crucial for the initial screening and identification of potential cardiac fibrosis biomarkers. At the same time, proteomics and peptidomics techniques are essential for validating these candidates and investigating their clinical applicability.^2,9,10^

Another important consideration is to assess whether the diagnostic and predictive capabilities of these biomarkers, as well as their cost-effectiveness in comparison with conventional biomarkers,^11^ are superior. Following this evaluation, it is crucial to propose new health technologies to ensure that advancements are effectively transferred to clinical practice for the benefit of patients.
